# Digital technologies for an improved management of respiratory allergic diseases: 10 years of clinical studies using an online platform for patients and physicians

**DOI:** 10.1186/s13052-020-00870-z

**Published:** 2020-07-25

**Authors:** Salvatore Tripodi, Andrea Giannone, Ifigenia Sfika, Simone Pelosi, Stephanie Dramburg, Annamaria Bianchi, Antonio Pizzulli, Jakob Florack, Valeria Villella, Ekaterina Potapova, Paolo Maria Matricardi

**Affiliations:** 1 Allergology Service Policlinico Casilino, Via Casilina, 1049, 00169 Rome, Italy; 2 TPS software solutions, Rome, Italy; 3 Pediatric Allergology Service Sandro Pertini Hospital, Rome, Italy; 4 Pediatric Pulmonology, Immunology and Intensive Care Medicine, Charité Universitätsmedizin Berlin, Berlin, Germany; 5 Pediatric Department, San Camillo Hospital, Rome, Italy; 6 Allergy Practice, Berlin, Germany

**Keywords:** Mobile health, E-diary, Pollen, Precision medicine, Digital

## Abstract

**Background:**

Digital health technologies carry the great potential of assisting physicians in making well-informed diagnostic and therapeutic decisions. In allergy care, electronic clinical diaries have been recently used to prospectively collect patient data and improve diagnostic precision.

**Objective:**

This review summarizes the clinical and scientific experience we gathered over 10 years of using a digital platform for patients suffering from seasonal allergic rhinitis.

**Methods:**

The mobile application and back-office of AllergyMonitor (TPS software production, Rome, Italy) enable patients to record their daily allergy symptoms as well as drug and immunotherapy intake plus possible side effects in a customizable way. The results can be accessed by the patient and attending physician as concise reports via a smartphone or computer. This technology has been used in several clinical studies and routine practice since 2009.

**Results:**

Our studies showed that A) the etiological diagnosis of SAR may be supported by matching prospectively registered symptoms with pollen counts; B) it is possible to perform a short-term prediction of SAR-symptoms at individual level; C) the adherence to daily symptom monitoring can remain high (> 80%) throughout several weeks when prescribed and thoroughly explained by the treating doctor; D) the use of mobile technology can improve adherence to symptomatic drugs as well as allergen-specific immunotherapy and E) the choice of the correct symptom-severity-score is critical at patient level, but not at group level.

**Conclusion:**

The studies and clinical practice based on the use of AllergyMonitor have proven the reliability and positive impact of a digital platform including an electronic diary (eDiary) on the diagnostic precision of SAR in poly-sensitized patients as well as patient adherence to both, drug therapy and allergen immunotherapy.

## Introduction

### Digital health

According to the World Health Organization (WHO), digital health or eHealth (short for “electronic health”) is defined as the cost-effective and secure use of information and communication technologies (ICTs) for health and health-related fields. mHealth (or “mobile health”), as a component of eHealth, involves the provision of health services and information via mobile technologies, such as mobile phones, tablet computers and Personal Digital Assistants (PDAs) [1]. As stated during the 71st World Health Assembly in Geneva (2018), mobile wireless technologies have the potential to revolutionize the interaction of citizens with national health services. The use of simple and easily accessible digital technologies can improve quality and coverage of care, increase the access to health information and services, raise awareness and promote positive changes in health behaviors to prevent the onset of acute and chronic diseases [2–4]. As the comprehensive implementation of digital health programmes forms a considerable challenge, the WHO Director General encouraged Member States to identify standardized approaches for applying digital health in their health systems and services. Several aspects of traditional health care will be changed by this digital health revolution: (a) the point of care will no longer be the clinic or laboratory, but the patient; (b) the approach to care will be based on the individual patient instead of patient groups with similar diseases; (c) the traditional hierarchy between doctor and patient (the former as an authority) based on prescriptions and orders will be replaced by a partnership-like collaboration (doctor as a guide); (d) patients’ data will be determined as personal property, not that of any institution; (e) decisions will be based on the analysis of big data sets in addition to the doctors’ experience; (f) the costs of care will be diminished [5].

### Digital health in Allergology

Digital Health may also have a very positive impact on the management of allergic patients. As stated in a position paper by the American College of Allergy, Asthma and Immunology (ACAAI), allergic patients benefit from telemedicine, for example through a better patient-doctor collaboration, easy access and adherence to allergists’ consultation as well as simplified prescription procedures. This positive impact is especially important for patients living in rural or remote areas. However, the authors also point out the need for improved regulations, certification programs, high attention to data protection, and the development of adequate reimbursement systems [6]. Recently, a Task Force of the European Academy of Allergy and Clinical Immunology (EAACI) published a position paper on “The Role of Mobile Health Technologies in Allergy Care” [7]. The study group examined over 130 allergy-related apps and reported on the role of mHealth technologies in the area of allergic rhinoconjunctivitis, asthma, atopic dermatitis, chronic urticaria as well as food allergies, anaphylaxis, drug, and venom allergies [7].

### Apps for allergic rhinitis

Although many apps are dedicated to the management and monitoring of allergic rhinitis, only few have been used in studies published in peer-reviewed international journals [7, 8]. A very large collaborative network focused on rhinitis and its treatment is accumulating evidence through the worldwide use of MASK-Air (MASK standing for Mobile Airways Sentinel Network). This electronic clinical diary assesses nasal, ocular and lung symptoms, as well as work impairment and global health via a visual analogue scale (VAS) [9]. MASK-Air has already accumulated real-life data from a large number of patients worldwide, whose analysis has led to innovative knowledge on productivity at work, innovative patterns of treatment, and new allergic disease phenotypes [10]. A model of individualized prediction of allergic rhinitis symptoms, named Patient’s Hay-fever Diary (PHD), has been developed in Austria [11]. By combining input from the patients (symptoms and medications) along with environmental information, an improved management of the disease is pursued through symptom forecasting [11].

In this review, we summarize the clinical and research experience that our group has gathered over the last decade with the platform “AllergyMonitor”, an eDiary for allergic rhinitis and allergen immunotherapy whose first version was developed in Rome, Italy, in 2009. In the following sections, we shall illustrate the structure and content of the digital platform, show exemplary reports of clinical cases, an illustration of the scientific studies based on AllergyMonitor, the perspective of studies in progress and the implications for allergy practice in real-life settings.

## AllergyMonitor

### AllergyMonitor: targets, structure, functions

**Fig. 1:**
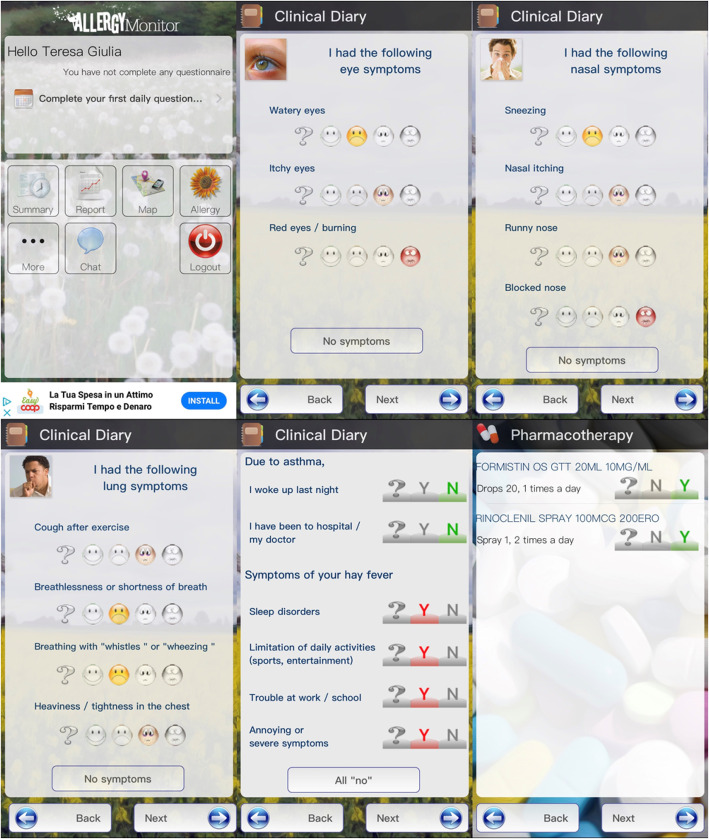
Screenshot examples of the front end of AllergyMonitor app. On a daily basis, the user fills a short and visually enhanced questionnaire about his symptoms of the eyes, nose and lungs, as well as a visual analogue scale on his/her general condition. Once activated by the doctor via the back-office, the user is also enabled to record his/her daily medication intake, adherence to sublingual immunotherapy and potentially occurring side effects. In order to provide a summary and feedback to the user, all entered data can be easily accessed within the app in summarized graphs showing the evolution of symptoms over time

Allergymonitor (TPS software production, Rome, Italy) is an online service developed in 2009 with the aim of enabling the recording of clinical symptoms, drug consumption and adherence to allergen-specific sublingual immunotherapy (SLIT) as well as monitoring efficacy of SLIT or subcutaneous immunotherapy (SCIT) by patients with allergic rhino-conjunctivitis and/or asthma. The system, available to everyone and straightforward to use, consists of two parts: a patient app (front end) and a website for the attending doctor (back-office), and the whole system is free during the actual Covid-19 pandemic. The app, that patients can freely download form Google Play and Apple Store, is available in different languages. On a daily basis, the user is requested to fill a short and visually enhanced questionnaire about his/her symptoms of the eyes, nose and lungs, as well as a visual analogue scale on his/her general allergic condition. Once activated by the doctor via the back-office, the app user is also enabled to record his/her daily medication intake, adherence to sublingual immunotherapy and potentially occurring side effects. In order to provide a summary and feedback to the user, all entered data can be easily accessed within the app in summarized graphs showing the evolution of symptoms over time (Fig. 1)**.**

**Fig. 2:**
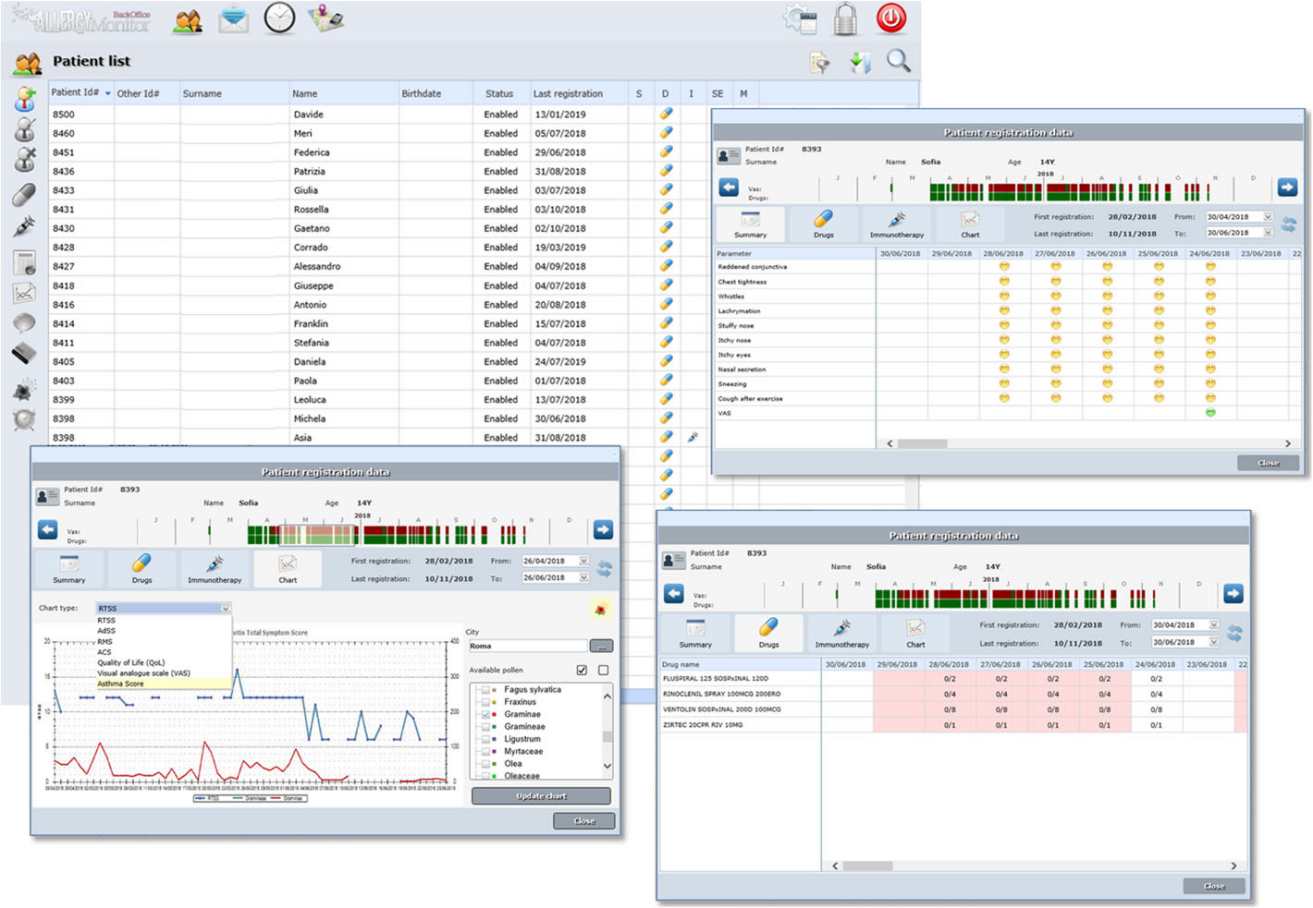
Screenshots of the doctor’s AllergyMonitor back-office. Via his/her back-office, the doctor is able to access a breakdown of all recorded data as well as individual patient reports accessible as different symptom (and medication) scores and matched to local pollen monitoring data. A messaging system between doctor and patient based on e-mail, chat or SMS (short message service) facilitates direct communication. The back-office enables the doctor to configure each patient’s front end individually by entering for example symptomatic drugs or adding an immunotherapy intake and side effects monitoring

Via his/her back-office, the doctor is able to access a breakdown of all recorded data as well as individual patient reports accessible as different symptom (and medication) scores (Rhinoconjunctivitis Total Symptom Score (RTSS), average adjusted symptom score (AdSS), rescue medication score (RMS), average combined score (ACS)) and matched to local pollen monitoring data, which is retrieved using validated methodologies. More specifically: pollen is collected in pollen traps, analyzed by aerobiologists, and this data is incorporated in AllergyMonitor. The data import is done by email, weekly through an automatic system in Italy, and manually for other countries where AllergyMonitor is being used. A messaging system between doctor and patient based on e-mail, chat or SMS (short message service) facilitates direct communication. An automatic alert system points out missed days of recording to both, front end and back-office users. Keeping in line with an approach of blended care, the back-office enables the doctor to configure each patient’s front end individually by entering for example symptomatic drugs or adding an immunotherapy intake and side effects monitoring (Fig. 2)**.**

### Clinical report

**Fig. 3:**
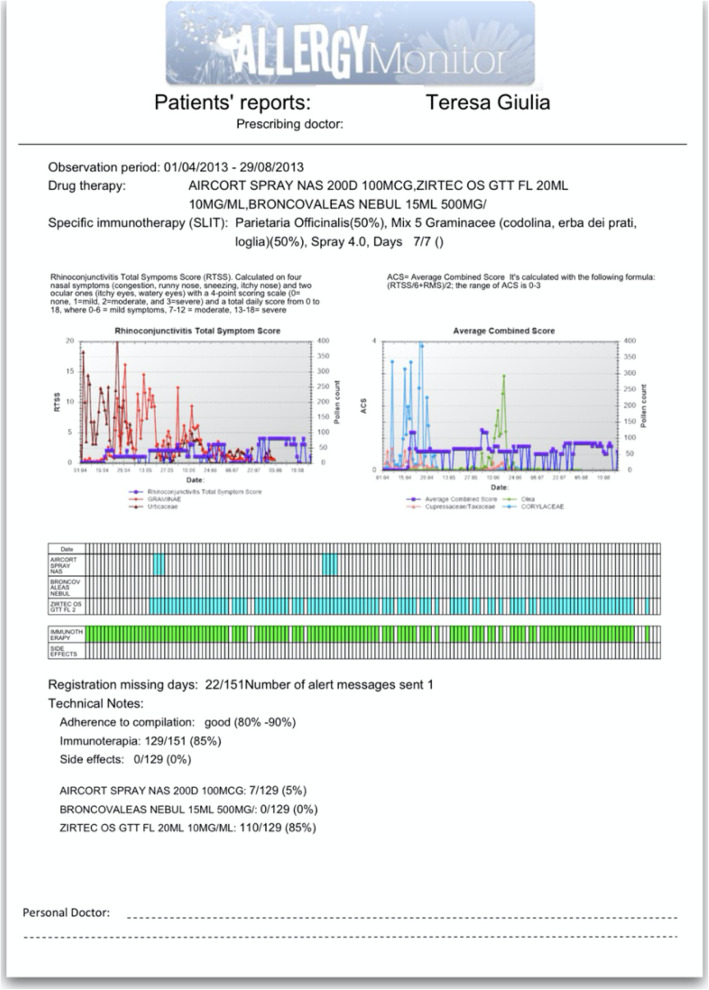
AllergyMonitor report: an example referring to a pediatric patient. The software generates a printable report for the user. The report is divided into several sections, as follows: **a**) doctor’s prescription: recommended monitoring period, pharmacotherapy, allergen-specific immune-therapy; **b**) symptoms vs pollen counts: graphs illustrating the time-trends of selected symptom severity scores and pollen counts; **c**) medication diary: table illustrating the intake of drugs and/or SLIT during the monitoring period; **d**) statistical summary: a series of indexes summarizing the patient’s adherence to symptom recording, as well as drug and SLIT intake; **e)** space for the doctor’s comments: marked empty space for comments and notes from the treating physician

On the basis of data registered via the front end (app), the software generates a printable report for the app user (Fig. 3)**.** The report is divided into several sections:

1. **Doctor’s prescription –** recommended monitoring period, pharmacotherapy, allergen-specific immunotherapy;
2. **Symptoms vs pollen counts -** Graphs illustrating the time-trends of selected symptom severity scores and pollen counts.
3. **Medication diary –** A table illustrating the intake of drugs and/or SLIT during the monitoring period.
4. **Statistical summary –** A series of indexes summarizing the patient’s adherence to symptom recording, as well as drug and SLIT intake.
5. **Space for the doctor’s comments –** marked empty space for comments and notes from the treating physician.

The report produced by AllergyMonitor can be printed and given by the patient to the physician of his/her choice, but also directly sent by mail from the app to the doctor. The doctor can then base further clinical examinations and diagnostic or therapeutic prescriptions also on the data prospectively acquired by the patient during the monitoring period. The volume, reliability and precision of the information provided by the eDiary may represent a valuable and time-efficient add-on to the information retrospectively collected during an often short interview, frequently done months after the relevant clinical episodes and possibly influenced by a strong recall bias.

### Scientific studies

Since 2010, our group has used AllergyMonitor in a series of clinical studies on allergic rhinitis. The results of these have illustrated how the system can be deployed to support the etiological diagnosis, symptom prediction, adherence to therapy, and decision on AIT prescription for patients presenting with seasonal allergic diseases.

**Fig. 4:**
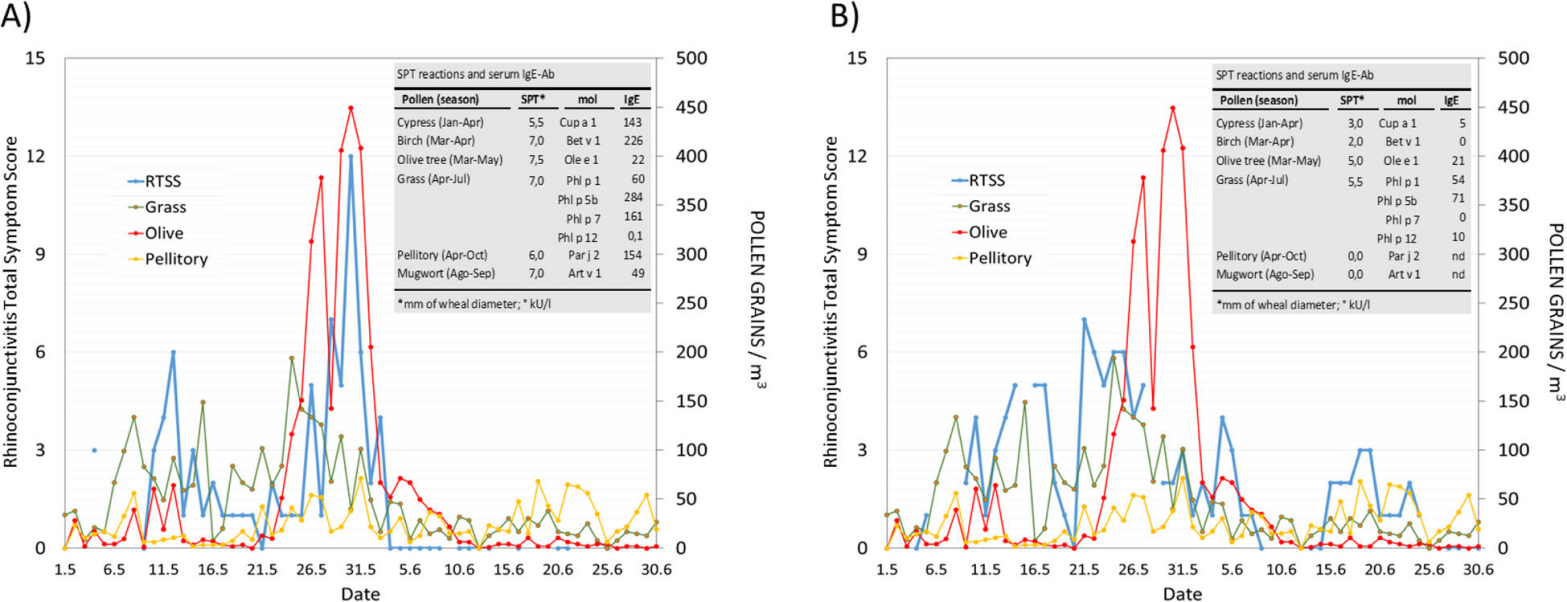
Trajectories of symptom severity vs pollen counts in two pediatric patients (**a**: patient 1; **b**: patient 2) from Ascoli Piceno with allergic rhinitis, and similar allergic profile, according to SPT and CRD. Data on severity of symptoms – collected with AllergyMonitor – have been reported as Rhinoconjunctivitis Total Symptom Score (RTSS). Pollen counts (grains/m3) were obtained from the local pollen trap. Reprinted with permission from [12]

**Fig. 5:**
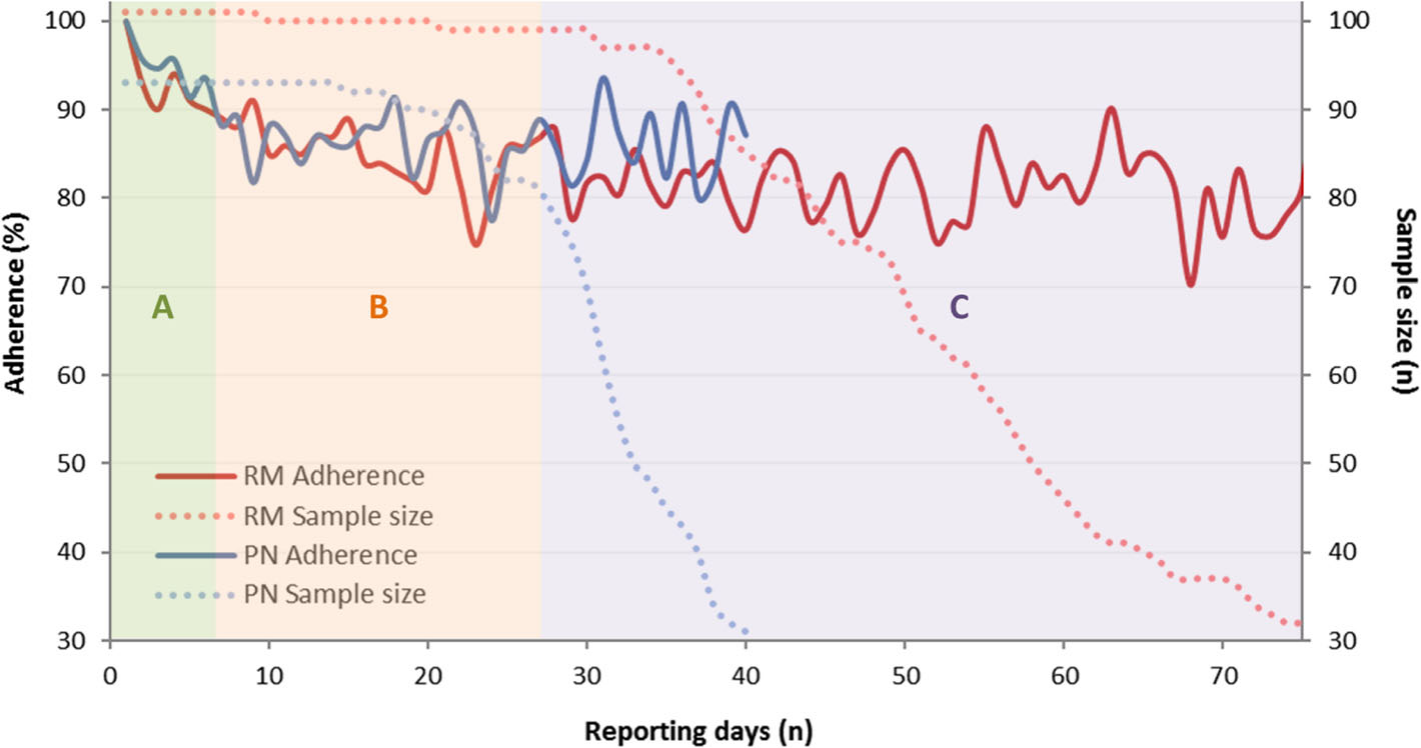
Adherence (%) by reporting day and study center. It is possible to describe three phases (indicated by light background color): the 1st phase (**a**), lasting 6 days, during which adherence falls from 100 to 90%; the 2nd phase (**b**), lasting approximately 20 days, during which adherence fluctuates until reaching 88%; the 3rd phase (**c**) during which it declines to 80%. Reprinted with permission from [22]

**Fig. 6:**
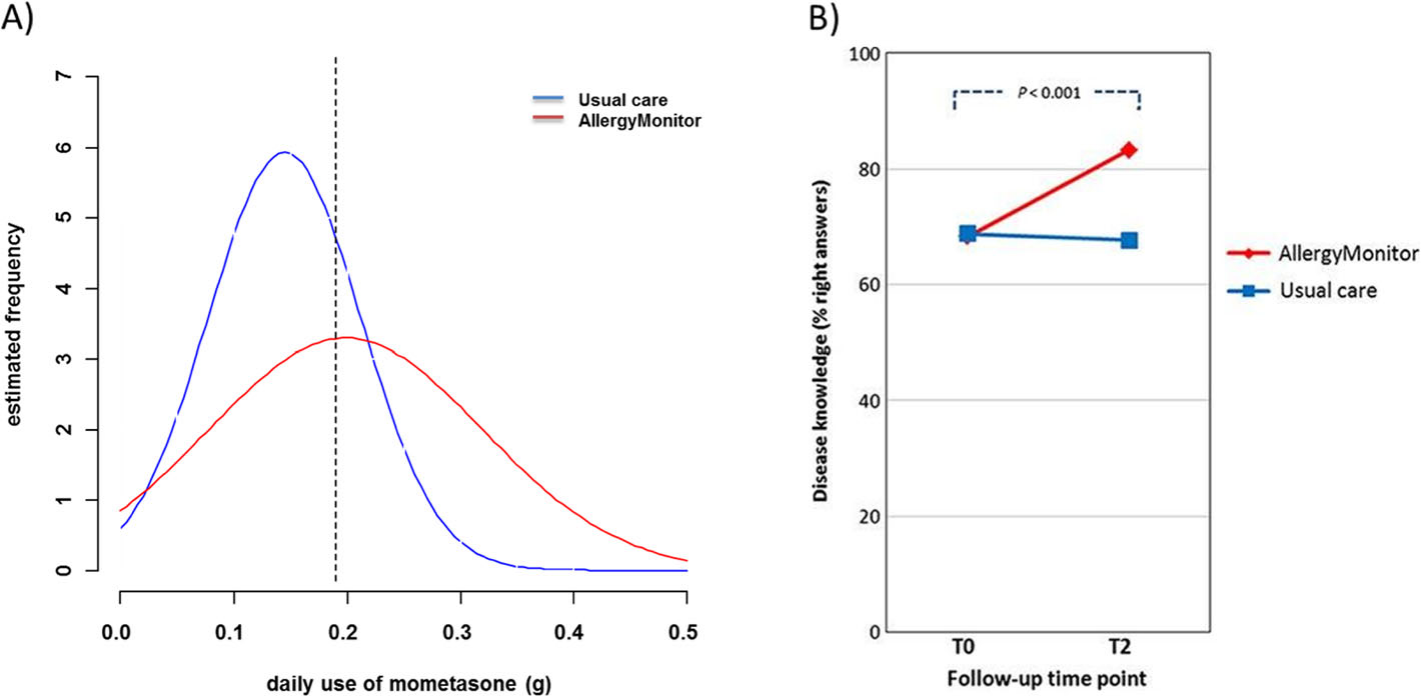
Impact of a eDiary on (**a**) medication adherence and (**b**) knowledge on disease. **a**) Adherence to daily medication with nasal corticosteroid (Mometasone) in children with Seasonal Allergic Rhinitis following usual care or being monitored with AllergyMonitor. **b**) Frequency of correct answers to knowledge test taken before and after the recording of symptoms connected to bits of information on allergic rhinoconjunctivitis provided via AllergyMonitor after every registration. Reprinted with permission from [15]

**Fig. 7:**
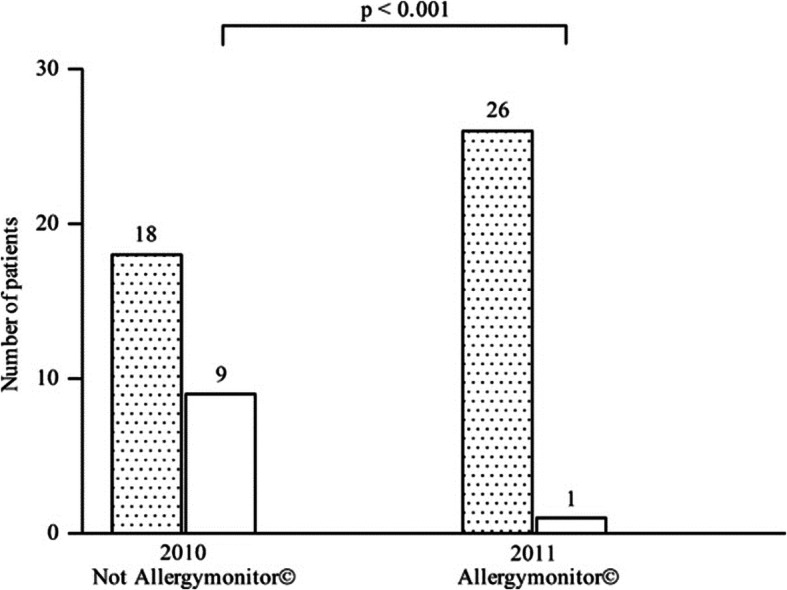
Impact of a eDiary on adherence to SLIT. Adherence to SLIT medication in children with Seasonal Allergic Rhinitis following usual care or being monitored with AllergyMonitor. Reprinted with permission from [26]

**Fig. 8:**
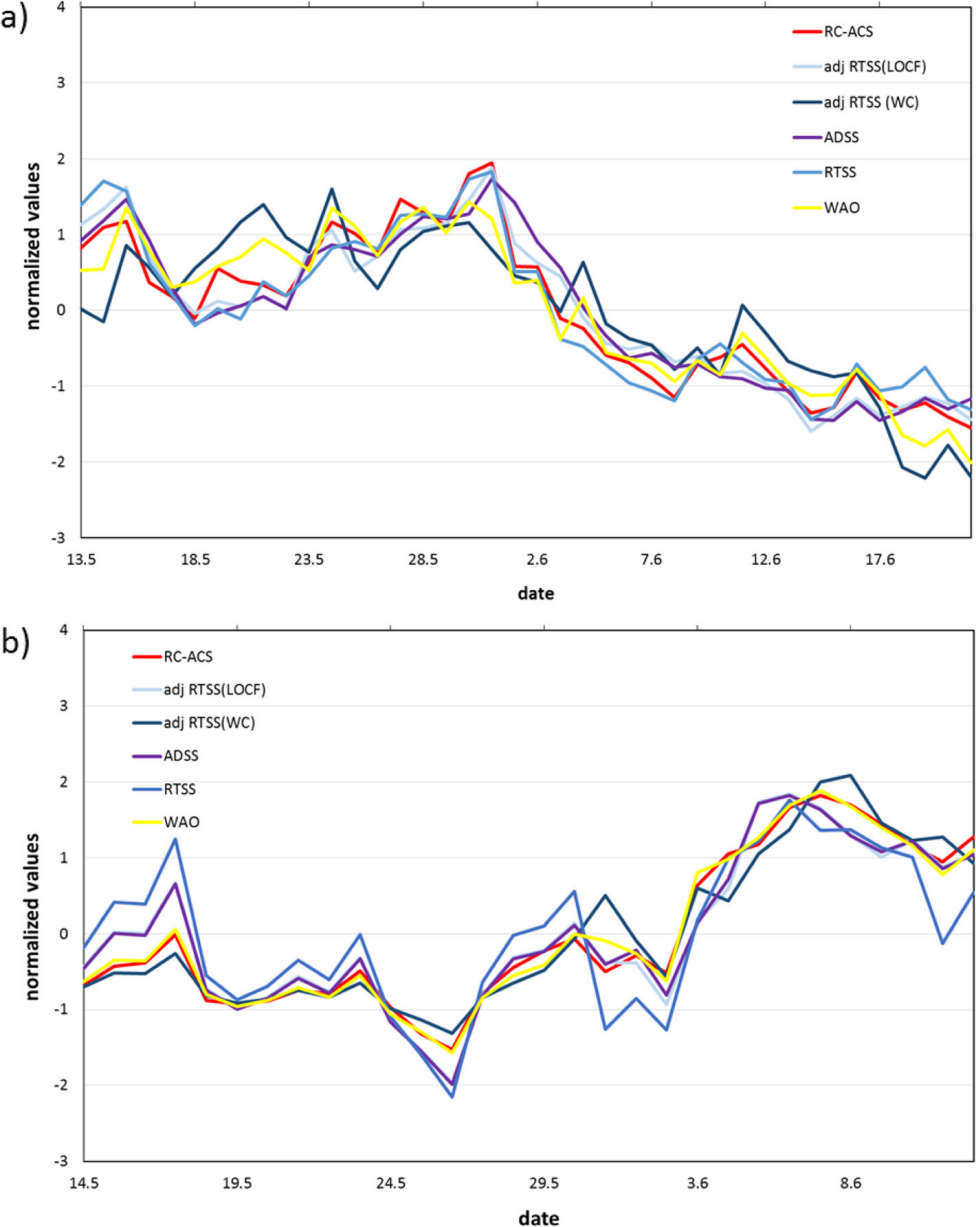
Parallel evaluation of multiple disease severity scores. Trajectories of normalized mean daily values of six disease severity scores in (**a**) 76 Italian, and (**b**) 29 German children with grass pollen-related seasonal allergic rhinitis, during the grass pollen season. Reprinted with permission from [14]

1. **Etiological diagnosis of seasonal allergic rhinitis (SAR) –** The analysis of the allergic rhinitis symptom severity scores during pollen exposure can be used to evaluate the clinical relevance of a patient’s sensitization to a specific pollen. This statement has been exemplified by describing two patients suffering from SAR with similar diagnostic challenges [12]. In both patients, no clear-cut decision could be reached based on a traditional allergological evaluation (clinical history, SPT,) plus molecular IgE assessment against relevant genuine and cross-reacting allergenic molecules (Ole e 1, Phl p1, Phl p 5). However, the prospective and consistent recording of nasal and conjunctival symptoms during the pollination period contributed fundamentally to the identification of the trigger-pollen (olive for patient one (Fig. 4a), grass pollen for patient two (Fig. 4b)). To our knowledge, this was the first report of an etiological diagnosis of pollen allergy substantiated by a smartphone app. The comparison of symptom severity scores (RTSS in this case) with pollen concentration data may therefore guide the doctor in the choice of the correct immunotherapy composition [12].
2. **Short-term prediction of allergic symptoms –** We used AllergyMonitor to test the efficiency of a model to forecast symptoms of pollen-related SAR at individual patient level. We analyzed prospectively recorded symptom and medication data (April to June 2010–2011) of 21 Italian children affected by allergic rhinoconjunctivitis. Using the average combined score (ACS) of symptoms and medication, we found that the short-term forecast of seasonal allergic rhinitis symptoms is possible even in highly poly-sensitized patients in geographic areas with complex pollen exposure. We further concluded that predictive models must be tailored to the individual patient’s allergic susceptibility. This may lead to a better use of anti-symptomatic drugs, especially considering their targeted intake before the expected raise of symptoms [13].
3. **Adherence to eDiary compilation -** Several e-Diaries are available for pollen allergies in European countries, some of them also having been used in trials or observational studies [10, 14–18]. In most of the study settings, the respective app was directly downloaded by patients, with no or only occasional intervention of the allergist [18–20]. Although the use of mobile technologies permits an unprecedentedly easy collection of big data sets independent from geographic location and social differences, some observational studies were characterized by a poor adherence of their users to data recording, sometimes even dropping below 10% after only 2 weeks [10, 19]. As the role of the attending physician has been shown to be of great importance for medication-compliance in patients [21], we wondered whether this also holds true for the adherence to digital symptom diaries. In an Italian bi-center study involving 101 children and 93 adults, patients were instructed very clearly on the use of AllergyMonitor and received personal reminders via phone in addition to automated alert messages in case of missed recording [22]. After completing the individualized monitoring periods, we could observe an overall adherence of ≥90% within the first week, with a decline to 80–90% between week 2 and 6 and then finally dropping to 70–80% after week 7 (Fig. 5). Interestingly, the individual adherence level in week 2 and 3 was able to predict a patient’s overall adherence to monitoring with enough confidence (Spearman’s *p* -0.55, *P* < .001 in both centers). We concluded that adherence to daily recording of an eDiary, provided that it is prescribed and eagerly motivated by a physician in a blended care setting, is very high.
4. **Adherence to drug therapy –** As an important cause for treatment failure in asthma and rhinitis is suboptimal adherence to local corticosteroids [23], we hypothesized that the use of a monitoring app with a reminder system might be able to optimize also medication-compliance and by this the clinical management of respiratory allergic diseases. The need to take medications regularly to obtain maximum effect even when asymptomatic is a particular problem for chronic diseases with episodic symptom occurrence, such as seasonal allergic rhinitis (hay fever). The reasons for suboptimal adherence are complex, but the key to successful management is good education both in the rationale for treatment and inhaler technique. Telemedicine has found its way into most corners of health care, but there is relatively little published on its potential role in allergic disease. Therefore, we have undertaken an original study looking at the value of telemonitoring on adherence to daily treatment with topical corticosteroids in children with severe hay fever [15]. The study demonstrated an improvement in both adherence to daily drug medication and disease knowledge. No improvement was seen in disease control, but pollen counts were low during the study period (Fig. 6).
5. **Adherence to Sublingual Immunotherapy –** The only disease-modifying treatment option for allergic rhinoconjunctivitis and asthma so far is an allergen-specific immunotherapy [24], which is mostly administered as repeated subcutaneous injections or the daily intake of sublingual tablets/drops. One of the most relevant problems linked to the long-term daily administration of sublingual immunotherapy (SLIT) is poor compliance and a high dropout rate. Only 50 and 20% of the patients starting the treatment with SLIT continue its daily administration in the second and third year of treatment, respectively [25]. When comparing long-term adherence of a small group of patients undergoing SLIT with usual care support versus a group of patients receiving SLIT plus digital adherence monitoring via AllergyMonitor, we observed a clear reduction in the drop-out rate in the second year of therapy among 28 patients with digital support [26]. (Fig. 7)
6. **Comparison of disease severity scores** – To assess the impact of different methodological approaches on the interpretation of digitally and prospectively collected data, we used several different symptom severity scores to analyze the data sets of two pediatric cohorts. In brief, 76 children with SAR from Ascoli Piceno (Italy) and 29 grass pollen allergic participants from Berlin (Germany), were asked to monitor their daily symptoms via the app during a period of 2 months within the local grass pollen season. We then prospectively compared six different severity scores for allergic rhinitis (AR) against pollen counts at both population and individual level (Fig. 8) [14], namely the Rhinoconjunctivitis Total Symptom Score (RTSS), the Adjusted Rhinoconjunctivitis Total Symptom Score (method: last observation carried forward) adjRTSS [LOCF], Adjusted Rhinoconjunctivitis Total Symptom Score (method: worst case) adjRTSS [WC] (rhinoconjunctivitis total symptom score [worst case]), the Rhino-conjunctivitis Allergy-Control-SCORE (RC-ACS©) the average combined score (ACS), and the average adjusted symptom score (AdSS). We found that the disease severity scores for SAR tend to provide similar results at population level but often produce heterogeneous slopes in individual patients. On this basis, we concluded that the choice of the disease severity score might have only a low impact on the outcome of a large clinical trial, but it may be crucial for the management of individual patients [14].

### Clinical routine and future perspectives

**Fig. 9:**
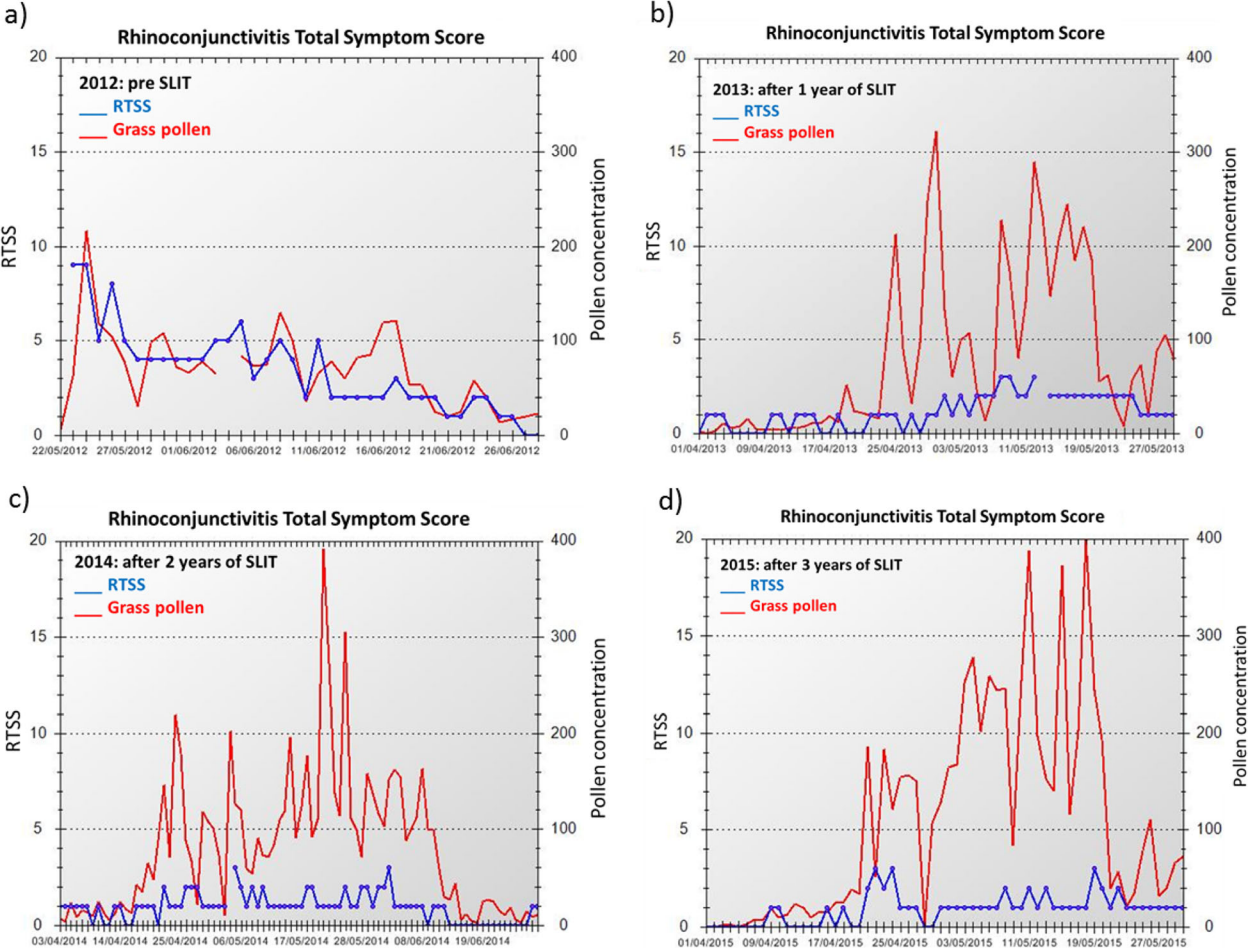
Symptom monitoring of a pediatric patient with SAR during grass pollen SLIT**.** RTSS and pollen trajectories before starting SLIT (**a**) and after 1 (**b**), 2 (**c**) and 3 years of SLIT (**d**)

The use of AllergyMonitor in routine clinical practice started in 2009 in the Pediatric Allergy Unit of the Pertini Hospital in Rome. During 10 years of activity, about 9500 patients seeking care for allergic rhinitis in this hospital have used the eDiary. On the other hand, about 130 allergists and pediatricians in 10 countries have prescribed the use of the app among their patients for clinical and/or research purposes. Individual user feedback from doctors shows, that the most appreciated benefits of prospectively collected clinical data plus pollen counts is the increased diagnostic precision especially for poly-sensitized patients but also the improved adherence to SLIT (Fig. 9). As one of the main benefits of AllergyMonitor is the interaction and feedback from the doctor, the initiative to introduce AllergyMonitor into the patients’routine comes predominantly from the medical teams.

To evaluate the combined impact of molecular IgE results and mobile health technologies on the precision of SAR diagnosis, our group has recently integrated AllergyMonitor in a more complex, still experimental clinical decision support system (CDSS). This CDSS is based on several steps of the diagnostic workup: collection of clinical history, retrospective pollen calendar, determination of allergic sensitization with allergen extracts, component-resolved diagnostics (that already has a very important role for the allergic diagnostic precision), clinical monitoring via eDiary, and parallel pollen count data. Algorithms then visualize the clinical and diagnostic picture of a patient by interpreting all entered data according to international guidelines for every individual step. The impact of this CDSS on the physicians’ diagnostic and therapeutic decisions has been evaluated in an Italian pilot study (@IT.2020) as well as an international multicenter project (@IT.2020MC) involving 815 patients and over 150 doctors from nine study centers in seven Southern European countries [27].

## Conclusions and perspectives

The studies and clinical practice based on the use of AllergyMonitor have proven the reliability of prospective digital data collection via eDiary as well as its impact on patient adherence to both, drug therapy and allergen immunotherapy. The role of the attending physician is fundamental, not only for an optimal adherence to digital technologies, but also in a collaborative setting of blended care. Over time, the interaction between doctors and patients will progressively change with the increasing use of digital opportunities. The possibility of expanding the use of eDiaries and other mHealth platforms into forecasting, through the translation of gathered data into a way of preventing individual patient exposure to unfavorable conditions such as high pollen counts, elevated air pollution levels, anti-symptomatic drug intake, is another important aspect to be taken into account. In order to implement these technologies responsibly in clinical practice to improve patient participation and care, studies and regulatory infrastructure are needed as acknowledged by international organizations such as the WHO. Given the current pandemic setting and unprecedent situation wordlwide, the impact and urgency of reliable and qualified mHealth systems is evident. In a time where human contact has been reduced, and health institutions and teams are overwelmed with critical patients, the benefits provided by digital platforms in patient care are substantial. It is, therefore, urgent to move forward with regulations and developments in this perspective.

## Data Availability

The datasets generated during and/or analyzed during the current study are not publicly available.

## Abbreviations

ACAAI: American College of Allergy, Asthma and Immunology
ACS: Average combined score
adjRTSS [LOCF]: Adjusted Rhinoconjunctivitis Total Symptom Score (method: last observation carried forward)
adjRTSS [WC]: Adjusted Rhinoconjunctivitis Total Symptom Score (method: worst case)
AdSS: Average adjusted symptom score
AIT: Allergen immunotherapy
AR: Allergic rhinitis
CDSS: Clinical decision support system
EAACI: European Academy of Allergy and Clinical Immunology
eDiary: Electronic diary
eHealth: Electronic health
ICT: Information and communication technologies
MASK: Mobile Airway Sentinel Network
mHealth: Mobile health
PDA: Personal digital assistant
RC-ACS©: Rhino-conjunctivitis Allergy-control-score
RMS: Rescue medication score
RTSS: Rhinoconjunctivitis total symptom score
SAR: Seasonal allergic rhinitis
SCIT: Subcutaneous immunotherapy
SLIT: Sublingual immunotherapy
SMS: Short message service
SPT: Skin prick test
VAS: Visual analogue scale
WHO: World Health Organization
